# Detection and Circulation of a Novel Rabbit Hemorrhagic Disease Virus in Australia

**DOI:** 10.3201/eid2401.170412

**Published:** 2018-01

**Authors:** Jackie E. Mahar, Andrew J. Read, Xingnian Gu, Nadya Urakova, Roslyn Mourant, Melissa Piper, Stéphanie Haboury, Edward C. Holmes, Tanja Strive, Robyn N. Hall

**Affiliations:** Commonwealth Scientific and Industrial Research Organisation (CSIRO), Acton, Australian Capital Territory, Australia (J.E. Mahar, N. Urakova, R. Mourant, M. Piper, S. Haboury, T. Strive, R.N. Hall);; The University of Sydney, Sydney, New South Wales, Australia (J.E. Mahar, E.C. Holmes);; Elizabeth Macarthur Agricultural Institute, Menangle, New South Wales, Australia (A.J. Read, X. Gu);; Invasive Animals Cooperative Research Centre, Bruce, Australian Capital Territory, Australia (N. Urakova, T. Strive, R.N. Hall)

**Keywords:** rabbit hemorrhagic disease virus, RHDV, RHDVa, rabbit hemorrhagic disease, RHD, biocontrol, recombinant, calicivirus, viruses, Australia

## Abstract

The highly virulent rabbit hemorrhagic disease virus (RHDV) has been widely used in Australia and New Zealand since the mid-1990s to control wild rabbits, an invasive vertebrate pest in these countries. In January 2014, an exotic RHDV was detected in Australia, and 8 additional outbreaks were reported in both domestic and wild rabbits in the 15 months following its detection. Full-length genomic analysis revealed that this virus is a recombinant containing an RHDVa capsid gene and nonstructural genes most closely related to nonpathogenic rabbit caliciviruses. Nationwide monitoring efforts need to be expanded to assess if the increasing number of different RHDV variants circulating in the Australian environment will affect biological control of rabbits. At the same time, updated vaccines and vaccination protocols are urgently needed to protect pet and farmed rabbits from these novel rabbit caliciviruses.

Rabbit hemorrhagic disease virus (RHDV) is a calicivirus in the genus *Lagovirus* causing acute hepatic necrosis in European rabbits (*Oryctolagus cuniculus*), leading to disseminated intravascular coagulation and death in >90% of susceptible animals (*1*). RHDV was first reported in China in 1984 and subsequently spread to South Korea by 1985, various European countries by 1986, and Mexico in 1988 (*1*). RHDV is now enzootic in domestic and wild rabbits in parts of Asia and Europe, and sporadic outbreaks continue to occur in the Americas, the Middle East, and Africa (*1*). RHDV also has had substantial ecological impacts in certain regions such as the Iberian Peninsula, where the rabbit is a keystone species, serving as a major prey species and actively altering the environment through grazing and burrowing behaviors (*2*).

Conversely, in Australia and New Zealand, the European rabbit is a major vertebrate pest, threatening the survival of native plants and animals, facilitating erosion through burrowing and grazing, and causing massive economic losses to agricultural sectors. In 1991, a strain of RHDV from Czechoslovakia (CAPM V-351) was imported into Australia for assessment of its suitability as a rabbit biocontrol agent; it has been used for this purpose since 1995, resulting in substantial benefits for both the economy and the environment (*3**–**5*). The same CAPM V-351 strain appeared in New Zealand in 1997, after it was presumably deliberately released by farmers (*6*). Circulating field strains (descendants of CAPM V-351) now cause regular natural outbreaks in both countries, and the original CAPM V-351 strain is continually rereleased through rabbit control programs (*7*).

RHDV has a 7.4-kb single-stranded positive-sense RNA genome (gRNA) comprising 2 open reading frames (ORFs) (*1*). ORF1 encodes a single polyprotein that is cleaved by the viral protease into 7 nonstructural proteins and the major capsid protein, viral protein (VP) 60, whereas ORF2 encodes a minor structural protein, (VP10) (Figure 1) (*1*). Ninety dimers of VP60 self-assemble to form the RHDV capsid, whereas VP10 is present in much smaller amounts, and its function is not well established (*1**,**8*). In addition to the gRNA, a 2.1-kb subgenomic RNA (sgRNA) can also be detected in virus preparations and encodes both structural proteins VP60 and VP10 (Figure 1) (*1*). The first 16 nt at the 5′ ends of the RHDV gRNA and sgRNA are identical, with the exception of 2 sites; this structure may facilitate template switching at the junction of RNA-dependent RNA polymerase (RdRp) and VP60 (*9*).

**Figure 1:**
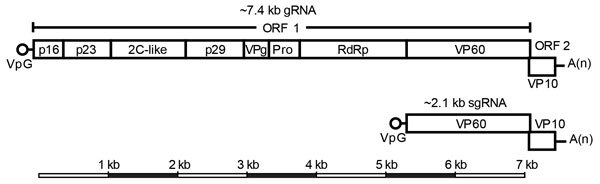
Genomic organization of RHDV. Top panel: RHDV has a polyadenylated single-stranded positive-sense gRNA of ≈7.4 kb consisting of 2 ORFs (open boxes), with the VP (open circle) covalently attached to the 5′ end. ORF1 encodes a polyprotein that is proteolytically cleaved to produce the major capsid protein, VP60, and the nonstructural proteins p16, p23, 2C-like protein (2C-like), p29, VPg, the viral protease, and the RdRp. ORF2 encodes the minor structural protein, VP10. Bottom panel: A 2.1-kb, VPg-linked, polyadenylated sgRNA is collinear with the 3′ end of the gRNA and encodes VP60 and VP10. High homology between the 5′ end of the sgRNA and the corresponding region of the gRNA (the RdRp and capsid junction) may facilitate template switching during replication. gRNA, RNA genome; ORF, open reading frame; Pro, protease; RHDV, rabbit hemorrhagic disease virus; RdRp, RNA-dependent RNA polymerase; sgRNA, subgenomic RNA; VP, viral protein; VPg, viral genome-linked protein.

RHDV strains are currently all classified as a single serotype, although phylogenetic analyses have led to the viruses being subclassified into 6 genogroups (*10**,**11*). The CAPM V-351 strain used for biocontrol in Australia and its descendants are genogroup G2 viruses. In the late 1990s, new genetically and antigenically distinct RHDV variants were reported and were designated as subtype RHDVa or genogroup G6 on the basis of differences in reactivity profiles with monoclonal antibodies and on sequence divergence (*10**,**12**,**13*). Despite this difference, vaccines developed for classic RHDV strains were still found to be protective against RHDVa when administered at the correct dose and within recommended intervals (*10**,**12**,**13*). RHDVa strains were first detected in Italy and were subsequently detected in multiple countries, including Portugal, the United States, China, Cuba, and South Korea (*12;* reviewed in *1*). In some geographic regions, it was suggested that RHDVa had replaced previous enzootic viruses (*14**–**17*).

Subsequently, a novel lagovirus, designated RHDV2, was reported from France in 2010 (*18*). This virus showed greater genetic and antigenic diversity than RHDVa compared with classic RHDV, with ≈82% nucleotide sequence similarity in VP60 between wild-type RHDV2 and RHDV (*11**,**18**,**19*). Early RHDV2 pathogenesis studies demonstrated that this virus had a longer disease duration, lower virulence, and lower mortality rates than RHDV and that it could cause disease in vaccinated rabbits (*18*). RHDV2 rapidly spread to Italy, Spain, Portugal, Great Britain, Scotland, the Azores, and Australia, and ongoing surveillance suggests that in many areas RHDV2 replaced existing circulating viruses, including RHDVa (*18**–**26*). RHDV2 has also been shown to cause disease in other lagomorph species, in contrast to RHDV and RHDVa, which are considered species-specific for the European rabbit (*27**–**30*).

In addition to the virulent RHDV, RHDVa, and RHDV2, several nonpathogenic lagoviruses have been described that are genetically distinct and have historically been called rabbit caliciviruses (RCVs). RCVs have been reported from various geographic locations; in contrast to the pathogenic hepatotropic lagoviruses, most RCVs mainly cause a nonclinical infection of the small intestine (*1*). Until 2014, only the benign rabbit calicivirus Australia-1 (RCV-A1) and the RHDV biocontrol strain CAPM V-351 (and its descendants) were circulating in Australia (*7**,**31*). In May 2015, RHDV2 was detected in Australia for the first time (*23*). Before the detection of RHDV2, another exotic RHDV incursion was reported to the World Organisation for Animal Health (OIE) in January 2014 (*32*). We describe the detection, spread, and evolution of this first exotic virus, RHDVa, in Australia during December 2013–May 2015, before the arrival of RHDV2.

## Materials and Methods

### Samples

Liver samples collected from rabbits suspected to have died from RHDV were submitted to the Elizabeth Macarthur Agricultural Institute (EMAI) (Menangle, New South Wales [NSW], Australia) or to CSIRO (Black Mountain, Australian Capital Territory [ACT], Australia) by veterinarians or NSW Local Land Service rangers. Samples were collected during December 1, 2013–April 30, 2015. No animal ethics permit is required in Australia for sampling of rabbits that are found dead.

For serologic analysis of healthy wild rabbits, we collected 20 wild rabbits from the ACT; these animals were shot from a vehicle using a 0.22-caliber rifle targeting the head or chest. We collected samples (blood, liver, duodenum, and bile) postmortem; all sampling was conducted according to the Australian Code for the Care and Use of Animals for Scientific Purposes as approved by the CSIRO Ecosystem Sciences animal ethics committee (approval #12–15).

### RHDV Antigen Capture ELISA

Liver samples submitted to EMAI were prepared by homogenizing ≈1 g of tissue in 5 mL of phosphate-buffered saline and centrifuging at 3,000 rpm for 10 minutes. Supernatant was tested in the RHDV antigen-capture ELISA as previously reported (*33*).

### Real-time Reverse Transcription PCR Assay

We screened samples for RHDV and RHDVa using real-time reverse transcription PCR (rRT-PCR). We applied sterile cotton swabs to the freshly cut surface of liver samples and transferred them to 5-mL vials containing 3 mL of sterile phosphate-buffered gelatin saline, then extracted viral RNA from 50-µL samples from these vials using the MagMAX-96 Viral Isolation System (Ambion, Austin, TX, USA) on a Kingfisher 96 magnetic particle handling system (ThermoFisher Scientific, Waltham, MA, USA).

We tested purified viral RNA (5 µL) in an RHDV-specific Taqman rRT-PCR assay using the AgPath-ID One-Step RT-PCR kit (ThermoFisher Scientific), based on detection of a conserved sequence of the VP60 gene with modifications (Table 1) (*34*). We developed an in-house Taqman rRT-PCR to specifically detect the exotic RHDVa virus (Table 1). Cycling conditions are available from the authors upon request.

**Table 1:** Primers and probes used for RHDV and RHDVa detection by real-time reverse transcription PCR in isolates from rabbits, Australia*

Name	Sense	Sequence, 5′ → 3′	Strain	Reference
vp60–7_FOR	+	ACY TGA CTG AAC TYA TTG ACG	RHDV	(*34*)
vp60–8_REV	–	TCA GAC ATA AGA AAA GCC ATT GG		(*34*)
vp60–9_FAM	Probe	CCA ARA GCA CRC TCG TGT TCA ACC T–FAM-BHQ1		Modified from (*34*)
RHDVXa2010-F1	+	GCACCCGGCAGTATTCTC	RHDVa	This study
RHDVXa2010-R1	–	CCCAGCCAGCGTACATCTG		This study
RHDVXa2010-P1	Probe	ACTGTCCAACACTCTCCACAGAACA–FAM-BHQ1		This study

*RHDV, rabbit hemorrhagic disease virus; vp, viral protein; +, positive; –, negative.

### Sequencing

We extracted RNA from 20–30 mg of liver tissue or bile using the RNeasy Mini Kit (QIAGEN, Hilden, Germany); the Maxwell 16 LEV simplyRNA Tissue Kit (Promega, Madison, WI, USA); or the Invitrogen Purelink viral RNA/DNA Mini Kit (ThermoFisher Scientific) following the manufacturers’ instructions. We performed first-strand cDNA synthesis from 5 μL of RNA with 500 ng of OligodT (18mer) (Geneworks, Thebarton, South Australia, Australia) using Invitrogen Superscript III (Life Technologies, Carlsbad, CA, USA) following the manufacturer’s directions. We conducted initial detection and typing of RHDV samples submitted to CSIRO (>1 isolate from each outbreak) using the universal lagovirus RT-PCR, as described previously (*35*), followed by Sanger sequencing of amplified products. RHDV G2 genomes were amplified as described previously (*7*). We amplified overlapping fragments of the RHDVa genome using Platinum Taq DNA polymerase high fidelity (Life Technologies) using primer sets detailed in Table 2 (cycling conditions available from the authors upon request). We purified PCR products using the QIAquick PCR purification kit (QIAGEN), quantified with the Qubit dsDNA BR assay (Life Technologies) and pooled in equimolar ratios.

**Table 2:** Primer sequences used for the amplification of RHDVa genomes for full-genome sequencing of isolates from rabbits, Australia*

Fragment	Primers	Sense	Sequence**, 5′ → 3′**	Reference
1	MRCV-F1	+	AAC TGC TAT TCT CCC AGA AAA GAA ACC CTT	This study
	RCVr1.2	–-	TGA GCT TSC CAG CDC CYT TCA TG	(*31*)
2	RCVf0.8	+	AAT GCT GTT GCT GTG GAY ACA AC	(*31*)
	RCVr3.3	–	GGR AGY CCY TCA TAG TCA TTG TCA T	(*31*)
3	RCVf3.0	+	GGY AAT GAY GAG TAT GAY GAG TGG CA	(*31*)
	RCVr4.7	–	ATR CCA CTT GGR AGY CCT CTT TTR G	(*31*)
4	Lago9	+	TGG NCC NAT YGC AGT YGG VRT TGA CAT GAC	(*36*)
	RCVr6.1	–	ACT ATC TGR CCR TTC CAY CTG TTG TC	(*31*)
5	Rab1b	+	CAG CDS GCA CTG CYA CCA CAG CAT C	(*35*)
	RHDV-12rev	–	ARC CTA ACT CAT ARG CCT GCA CAG TCG	(*37*)
6	RHDVf2	+	GTT TTG GTA CGC TAA TGC TGG ATC TGC	(*37*)
	RHDV_end	–	TTT TTT TTT TTT TTT TTT TTT TTT TTT TTA TAG CTT ACT TTA AAC TAT AAA CCC AAT TAA ACC	(*38*)

*MRCV, Michigan rabbit calicivirus; RCV, rabbit calicivirus; RHDV, rabbit hemorrhagic disease virus; +, positive; –, negative.

### Library Preparation and Illumina Sequencing

We prepared DNA libraries of PCR amplicons using the Nextera XT DNA Sample Prep Kit (Illumina, San Diego, CA, USA). Sequencing was performed on an Illumina MiSeq using the 300 cycle paired-end MiSeq Reagent Kit v2 (Illumina), as described previously (*7*).

### NGS Genome Assembly and Data Analyses

We performed read quality assessment and trimming and merged overlapping paired-end reads, as described previously (*7*). We then mapped individual cleaned reads to the RHDV reference genome (Genbank accession no. M67473.1) using the Geneious in-built mapper tool as available in Geneious version 8.1.6 (*39*) and generated a majority consensus sequence. Primer sequences were trimmed from the 5′ and 3′ genomic termini. We named sequences using the syntax country/state/isolate identifier/date, with date in the format yyyy/mm (e.g., AUS/NSW/BER-1/2013/12). Consensus sequences were aligned with representative RHDV, RHDV2, and RCV-A1 sequences for recombination and phylogenetic analyses. We submitted all sequences generated in this study to GenBank (accession nos. KY628306–21.

We inferred maximum likelihood phylogenies for both the nonstructural genes (44 sequences, 5,238 nt) and the VP60 gene (47 sequences, 1,743 nt) using PhyML (*40*). We estimated trees using the general time reversible model with rate heterogeneity among sites (4 discrete rate categories) and a proportion of invariant sites (determined as most appropriate using jModel test [*41*]), with a combination of nearest neighbor interchange and subtree pruning and regrafting branch swapping methods employed to search for the optimal topology. We rooted trees using a European brown hare syndrome virus sequence (EBHSV; GenBank accession no. Z69620) and estimated branch support using 1,000 bootstrap replicates.

We screened a full genome alignment containing the 15 newly acquired sequences and 12 reference genomes (27 sequences, 7,303 nt) for recombination using the RDP, GENECONV, MaxChi, and BootScan methods available in the RDP4 package (*42*), with p<0.05 representing significant evidence of recombination. We confirmed recombination events by phylogenetic analyses on either side of the proposed breakpoint.

### Serologic Analysis

We tested serum specimens from the shot wild rabbits for the presence of RCV-A1 antibodies using a specific blocking ELISA (*43*). We also tested for antibodies against RHDV using a competition ELISA and used ELISAs for RHDV IgA and IgM subclass antibodies as described previously (*44*).

## Results

In late December 2013 and January 2014, a property in northern Sydney, NSW, Australia, experienced the sudden deaths of 30 domestic rabbits from a population of 80. The treating veterinarian submitted liver samples to EMAI for routine RHDV testing in January 2014. The samples showed little or no reactivity in the RHDV antigen capture ELISA but tested strongly positive in the RHDV-specific rRT-PCR. This result was in contrast to the typical reactivity of field strains of RHDV from Australia, which react strongly in both the RHDV antigen capture ELISA and the RHDV-specific rRT-PCR (Table 3). To further characterize this calicivirus, a 326 bp fragment of the VP60 capsid gene was amplified using a universal lagovirus PCR (*35*). Sanger sequencing of this fragment identified the virus as a member of the RHDVa group (RHDV G6), a virus not previously reported in Australia.

**Table 3:** Cross-reactivity of different diagnostic tests used to identify novel RHDVa variant in rabbits, Australia*

Test	RHDV	RHDVa	RCV-A1
RHDV antigen capture ELISA	+++	–/+	–
RCV-A1 blocking ELISA	–	–	+++
RHDV competition ELISA	+++	++	–/+
RHDV IgA ELISA	+++	+++	++
RHDV IgM ELISA	+++	+++	++
VP60 rRT-PCR	+++	+++	+++
RHDVXa-2010 rRT-PCR	–	+++	–
Lagovirus RT-PCR	+++	+++	+++

*All tests used the VP60 capsid protein coding region as target region. RCV, rabbit calicivirus; RHDV, rabbit hemorrhagic disease virus; rRT-PCR, real-time reverse transcription PCR; VP, viral protein; –, no cross-reactivity; –/+, minimal cross-reactivity; ++, moderate cross-reactivity; +++, marked cross-reactivity.

**Figure 2:**
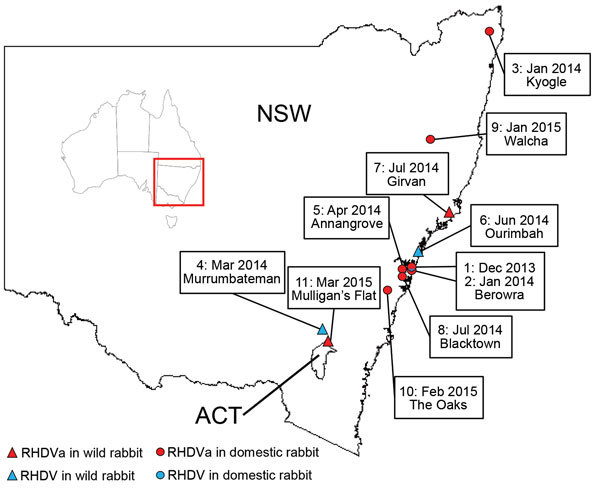
RHDV and RHDVa detections in eastern Australia, January 2014–March 2015. Sites where RHDVa and Australian RHDV field strains were detected are indicated on the map and numbered according to the order in which the outbreaks occurred. Inset shows location of NSW and ACT in Australia. ACT, Australian Capital Territory; NSW, New South Wales.

From January 2014 through March 2015, the exotic RHDVa strain was detected by rRT-PCR in 7 reported outbreaks in domestic rabbit breeding facilities (outbreaks 1, 2, 3, 5, 8, 9, and 10; Table 4; Figure 2), as well as in wild rabbits from 2 locations (outbreaks 7 and 11; Table 4; Figure 2). Overall, >70 rabbit deaths were reported in domestic rabbits from properties where RHDVa was confirmed.

**Table 4:** Outbreaks of RHDV in rabbits, Australia, January 2014–March 2015*

Outbreak no.	Isolate name	Collection date	Location, state	Variant	Rabbit origin	GenBank accession no.
1	AUS/NSW/BER-1/2013/12†	Dec 2013‡	Berowra, NSW	RHDV G2	Domestic	KY628307
1	AUS/NSW/BER-2/2013/12†	Dec 2013‡	Berowra, NSW	RHDVa	Domestic	KY628309
2	AUS/NSW/BER-3/2014/01†	Jan 2014	Berowra, NSW	RHDVa	Domestic	KY628310
2	AUS/NSW/BER-4/2014/01†	Jan 2014	Berowra, NSW	RHDVa	Domestic	KY628311
2	AUS/NSW/BER-5/2014/01	Jan 2014	Berowra, NSW	RHDVa	Domestic	NA
2	AUS/NSW/BER-6/2014/01	Jan 2014	Berowra, NSW	RHDVa	Domestic	NA
2	AUS/NSW/BER-7/2014/01	Jan 2014	Berowra, NSW	RHDVa	Domestic	NA
2	AUS/NSW/BER-8/2014/01†	Jan 2014	Berowra, NSW	RHDVa	Domestic	KY628312
2	AUS/NSW/BER-9/2014/01	Jan 2014	Berowra, NSW	RHDVa	Domestic	NA
2	AUS/NSW/BER-10/2014/01†	Jan 2014	Berowra, NSW	RHDVa	Domestic	KY628308
2	AUS/NSW/BER-11/2014/01	Jan 2014	Berowra, NSW	RHDVa	Domestic	NA
3	AUS/NSW/KYO-1/2014/01†	Jan 2014	Kyogle, NSW	RHDVa	Domestic	KY628316
3	AUS/NSW/KYO-2/2014/01	Jan 2014	Kyogle, NSW	RHDVa	Domestic	NA
4	AUS/NSW/BlueGums1/2014/03	Mar 2014	Murrumbateman, NSW	RHDV G2	Wild	KT006732.1
5	AUS/NSW/ANN-1/2014/04†	Apr 2014	Annangrove, NSW	RHDVa	Domestic	KY628306
6	AUS/NSW/OUR-1/2014/06†	June 2014	Ourimbah, NSW	RHDV G2	Wild	KY628318
6	AUS/NSW/OUR-2/2014/06†	June 2014	Ourimbah, NSW	RHDV G2	Wild	KY628319
7	AUS/NSW/GIR-1/2014/07†	July 2014	Girvan, NSW	RHDVa	Wild	KY628314
7	AUS/NSW/GIR-2/2014/07†	July 2014	Girvan, NSW	RHDVa	Wild	KY628315
8	AUS/NSW/BLA-1/2014/07†	July 2014	Blacktown, NSW	RHDVa	Domestic	KY628313
9	AUS/NSW/WAL-1/2015/01†	Jan 2015	Walcha, NSW	RHDVa	Domestic	KY628320
10	AUS/NSW/OAK-1/2015/02†	Feb 2015	The Oaks, NSW	RHDVa	Domestic	KY628317
11	AUS/ACT/MF-109/2015/03	Mar 2015	Mulligan’s Flat, ACT	RHDVa	Wild§	KY628321

*Samples are numbered according to the date on which they were collected. These numbers correspond to those used on the map in Figure 2. ACT, Australian Capital Territory; NSW, New South Wales; RHDV, rabbit hemorrhagic disease virus. †Samples for which the full genome was sequenced in this study. **‡**Detection of RHDVa confirmed in January 2014. **§**Virus sequence obtained from a healthy shot rabbit.

Outbreak 11, from Mulligan’s Flat Nature Reserve near Canberra, ACT, Australia, was suspected when rangers detected a sudden reduction in rabbit numbers during their rabbit eradication program, although no rabbit carcasses were recovered. Subsequent serologic analysis of 20 healthy shot rabbits revealed a high proportion of rabbits positive for RHDV-specific IgM (7/20) and IgA (15/20), indicating a recent virus outbreak (*45*). However, despite moderate to high IgA and IgM titers, the serum specimens tested low or negative in the RHDV competition ELISA and RCV-A1 blocking ELISA, which are known to be more strain-specific than the IgM and IgA ELISAs (data not shown). This finding indicated possible exposure of the Mulligan’s Flat rabbit population to a lagovirus that was antigenically distinct from both RHDV and RCV-A1. Because RHDV RNA can be detected in the bile of recovering animals for as long as 15 weeks postinfection (*34*), we extracted RNA from the bile of IgM-positive individuals and analyzed it using the universal lagovirus PCR (*35*). We amplified a lagovirus-specific fragment from 1 animal (AUS/ACT/MF-109/2015/03; Table 4), and Sanger sequencing confirmed the presence of the exotic RHDVa in this rabbit.

We also detected Australian field strains (RHDV G2) during the same sampling period on 3 occasions, 2 from wild rabbits (outbreaks 4 and 6; Table 4), and 1 from a domestic rabbit facility (outbreak 1; Table 4). The strain from the domestic rabbit facility was identified in samples from the first reported outbreak of the exotic RHDVa, indicating that both viruses were active simultaneously on this property.

**Figure 3:**
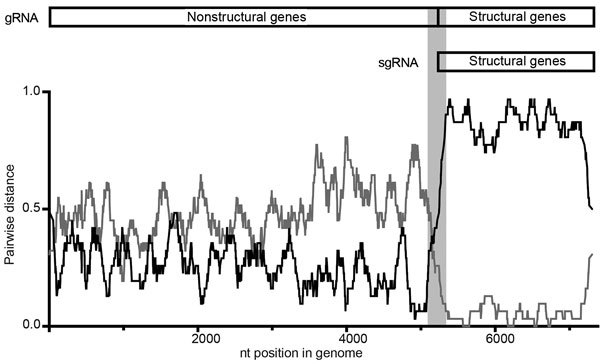
Recombination detection program plot (*42*) demonstrating recombination in a representative rabbit hemorraghic disease virus type a (RHDVa) strain from Australia. The pairwise identity of the recombinant, KYO-1, with the putative parental strains, RHDVa/AB300693.2/JPN/Hokkaido/2002 (black) and RCV-A1/EU871528.1/AUS/MIC-07(1–4)/2007 (dark gray), is plotted according to genome position (nt). A clear crossover event can be observed at the junction of RNA-dependent RNA polymerase and viral protein 60. The window size was set to 30. A schematic representation of the rabbit lagovirus gRNA and sgRNA is shown above the RDP plot to illustrate the genomic structure. The light gray bar shows the region where recombination was detected. gRNA, RNA genome; RHDV, RCV, rabbit calicivirus; sgRNA, subgenomic RNA.

**Figure 4:**
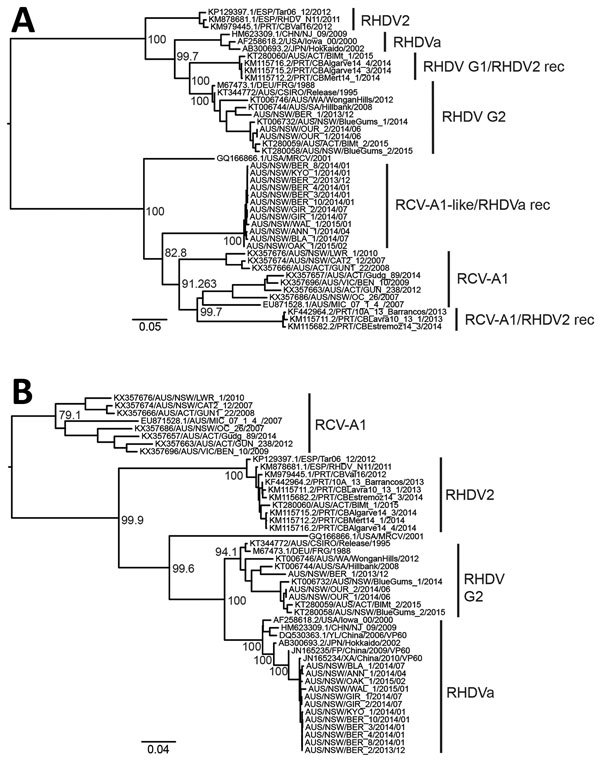
Phylogenetic analysis of viral protein 60 (VP60) capsid (n = 47) and nonstructural (n = 44) genes of RHDV strains from Australia and reference sequences. Maximum likelihood phylogenies of the A) VP60 capsid genes and B) nonstructural genes were prepared from an alignment of the newly sequenced RHDV samples (bold) along with published sequences (accession numbers of published sequences indicated in the taxa name). The JN165235/FP/China/2009 and JN165234/XA/China/2010 sequences were restricted to the capsid gene tree because nonstructural gene sequences are not available for these viruses. Variant names for each cluster are indicated. Recombinant (rec) variants are labeled as nonstructural/capsid gene type. Phylogenies were rooted using an early European brown hare syndrome virus isolate (not shown). Bootstrap support values are shown for the major nodes. Scale bars indicate nucleotide substitutions per site. RCV, rabbit calicivirus; RHDV, rabbit hemorrhagic disease virus.

We conducted full-genome sequencing for 15 RHDV isolates we sampled during December 2013–March 2015. We could not recover a full-length genome from AUS/ACT/MF-109/2015/03. Recombination and phylogenetic analyses of the nonstructural genes indicated that all RHDVa viruses were recombinants between RHDVa and an RCV-A1-like virus, with strong bootstrap support (Figures 3, 4). Specifically, we found strong evidence for recombination with a putative breakpoint located between the RdRp and VP60 genes (genome position 5,304 in Genbank accession no. M67473.1 sequence numbering), detected by all recombination detection methods in RDP4 (Figure 3). Phylogenetic analysis of the complete VP60 capsid gene revealed that the RHDV G2 viruses clustered with other recent RHDV G2 samples from Australia and that the RHDVa samples clustered (99% nucleotide sequence identity) with exotic RHDVa variants, particularly a variant from China, XA/China/2010 (Genbank accession no. JN165234) (Figure 4, panel A) (*14*). In contrast, the nonstructural genes of the RHDVa viruses found in Australia clustered with the RCV-A1–like clade that contains several other lagoviruses, including RCV-A1/RHDV2 recombinant viruses (*46*) and Michigan RCV (*47*), neither of which have been detected in Australia (Figure 4, panel B). Although the nonstructural genes of these viruses cluster most closely with RCV-A1, they are quite distinct, sharing only 83.3%–84.4% nucleotide identity with RCV-A1 sequences in this region, and could therefore represent a novel lagovirus genotype.

## Discussion

We describe the detection and characterization of RHDVa in Australia, the first of 2 recent incursions of exotic lagoviruses (*23*). The route of entry of these viruses into Australia is unclear. The spread of RHDVa from Europe to distant locations such as Asia, the Americas, Reunion Island, and Australia (*1*) highlights the ease with which this virus can be disseminated, despite strict quarantine regulations. Caliciviruses are known to be highly environmentally stable, they replicate to very high titers in infected rabbits, and they are efficiently transmitted (*1*), suggesting that they would be relatively easy to disseminate inadvertently, analogous to the situation observed with the emergence of canine parvovirus (*48*).

Unexpectedly, full-genome sequencing revealed that all RHDVa viruses in Australia had recombinant genomes, with the capsid gene closely related to a 2010 virus from China and nonstructural genes related to RCV-A1–like viruses (Figure 4). This finding highlights the importance of recombination in the generation of genetic diversity in this virus family. The emergence of this RCV-A1–like/RHDVa recombinant is of particular interest given the differences in tissue tropisms of RHDVa, which replicates in the liver, and nonpathogenic RCVs, which replicate in the small intestine (*1**,**35*). Recombinant viruses containing RCV-A1–like nonstructural genes and RHDV2 structural genes have also been reported (*46*). Although RHDV is widely disseminated in an infected animal, there is no evidence to suggest it can actively replicate in the small intestine. Conversely, RCV-A1 has only been shown to infect the columnar epithelial cells of the small intestine (*35*). Therefore, we hypothesize that the recombination event likely occurred during early viral replication in macrophages, which has been suggested for both RHDV (*1*) and RCV-A1 (T. Strive, unpub. data). Although the exact parental virus and source of the RCV-A1–like nonstructural genes were not identified, the distance between the nonstructural genes of RHDVa in Australia and currently sampled RCV-A1 nonstructural genes (83.3%–84.4% nt identity, Figure 4B) suggests either that the recombination event occurred before the virus arrived in Australia with an as-yet uncharacterized calicivirus or that recombination did occur in Australia but the parental virus belongs to a divergent RCV-A1 lineage that has not yet been identified.

The exotic RHDVa variant was first detected in Australia in samples from an RHD outbreak in December 2013 in domestic rabbits in a suburb of northern Sydney (*32*). During January 2014–March 2015, this virus was detected in a further 8 outbreaks. Detections were localized to southeastern Australia, predominantly in the Sydney basin. In contrast, the initial spread of the CAPM V-351 virus in 1995 was estimated to be 50 km/wk (*1*). Seven of the 9 documented RHDVa outbreaks were in domestic rabbits, although this probably reflects sampling bias, because very limited surveillance occurred in NSW and ACT in wild rabbits during the sampling period. 

The seroprevalence of RHDV and RCV-A1 antibodies was shown to be very high in wild rabbit populations in southeastern Australia (*49*), which may limit RHDVa infection and transmission rates in wild rabbits compared with unvaccinated domestic rabbits, due to partial or complete immunological cross-protection from previous infection with RHDV, RCV-A1, or both. In addition, RHDV outbreaks are much more readily detected in domestic rabbit facilities where animals are checked daily, whereas outbreaks in wild rabbits often go unnoticed, particularly as carcasses are rapidly removed by scavengers. Movement of domestic rabbits for breeding and showing purposes may also have facilitated spread of the virus in this population within the Sydney basin. Nevertheless, RHDVa was confirmed in 2 wild rabbit populations, in Girvan, NSW (outbreak 7), and Mulligan’s Flat (outbreak 11; Table 4), suggesting infection and transmission of RHDVa was occurring in wild populations.

In Mulligan’s Flat (outbreak 11), we recovered RHDVa RNA from a healthy shot rabbit following an observed decline in rabbit numbers. Subsequent serologic analyses from a sample of healthy shot rabbits suggests that high levels of cross-reacting isotype antibodies (IgA, IgM, and IgG), in combination with low or negative test results for specific competition ELISAs for RHDV and RCV-A1, may be used as a tool to infer previous exposure of a rabbit population to RHDVa. This particular serologic profile may prove useful for large-scale field epidemiology studies attempting to track the spread and impact of RHDVa in the Australian landscape, even in the absence of a specific serologic test. Furthermore, our study demonstrates that PCR analysis of bile in recovering, recently RHDV-infected (IgM-positive) rabbits can be an additional tool to determine to which virus a wild rabbit population had been exposed.

Several of the RHDVa cases in domestic rabbits were reported to have occurred in previously vaccinated rabbits, although details about the vaccination regimens and intervals were difficult to retrieve. The RHDVa variant JN165234/XA/China/2010, which closely resembles the VP60 sequence of the RHDVa from Australia we describe, was reported to be able to partially overcome protection induced by vaccination against classic RHDV (*14*). However, the vaccine used in that study was not a commercial preparation, and dose rates were not provided (*14*). The same study demonstrated that vaccination against classic RHDV induced 100% protection against another RHDVa virus from China (*14*). RHDV2 is considered to be a second lagovirus serotype, but the OIE Terrestrial Manual does not discriminate between RHDV and RHDVa with respect to vaccination and conservatively recommends that breeder rabbits be vaccinated every 6 months to ensure protection in commercial rabbitries (*11*). Australian authorities have issued updated vaccination recommendations subsequent to the detection of RHDV2 to reflect those of the OIE (*11*). Ultimately, a polyvalent vaccine effective against all viruses circulating in Australia (RHDV and RHDV2) would be preferred for domestic rabbits.

Before the detection of RHDVa, the deliberately released CAPM V-351 and its descendants were the only RHDV variants circulating in Australia, despite both RHDVa and RHDV2 circulating overseas since 1997 and 2010, respectively (*7**,**12**,**18*). As such, the RHDV antigen capture ELISA was routinely used for diagnostic purposes. Given that the RHDVa virus we report here shows little to no reactivity in this ELISA, it is recommended that the monoclonal antibodies used in diagnostic ELISAs be updated, as described in the OIE Terrestrial Manual, or that alternative detection methods such as RT-PCR be used for RHD diagnosis in Australia (*11*). At this stage, it is unclear what effects the presence of this new virus will have on Australia’s wild rabbit populations and on biocontrol efforts using RHDV. With the recent (March 2017) release of another RHDVa virus (an RHDVa from South Korea), it is critical that ongoing monitoring and surveillance systems are in place to explore the interactions of these new viruses as they establish under Australian field conditions (*50*). In this context, it will be particularly interesting to determine if the recombinant RHDVa described here persists in the face of the exotic incursion of RHDV2, which has reportedly replaced RHDV and RHDVa viruses in many parts of Europe (*24**–**26*). Once again, the epidemiology of these viruses demonstrates the utility of Australia’s rabbit populations serving as a model system for disease emergence, viral competition, and evolution.
