# Correction: Vol. 24, No. 1

**DOI:** 10.3201/eid2403.C12403

**Published:** 2018-03

**Keywords:** errata, erratum, correction

Figure 4 and part of the legend for Figure 1 were incorrect in Detection and Circulation of a Novel Rabbit Hemorrhagic Disease Virus in Australia (J.E. Mahar**Jackie E. MaharJackie E. Mahar**et al.). The article has been corrected online (https://wwwnc.cdc.gov/eid/article/24/1/17-0412_article).

**Figure 4:**
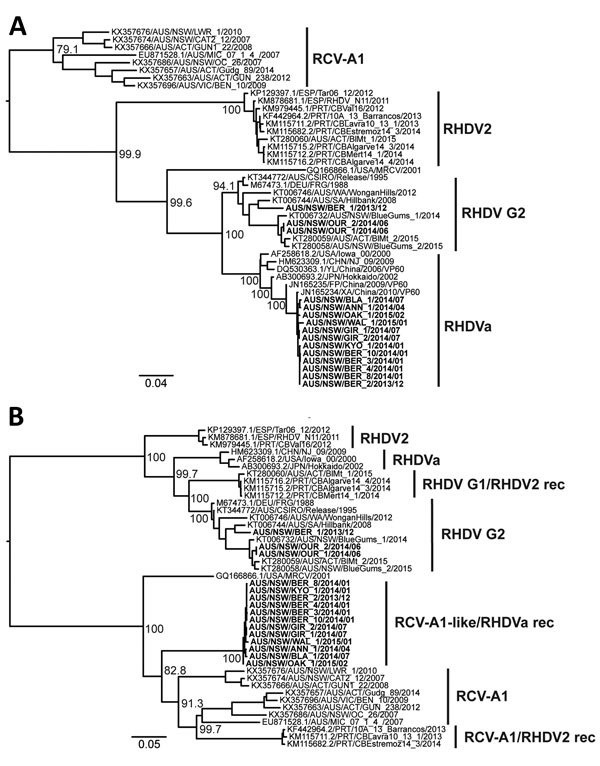
Phylogenetic analysis of viral protein 60 (VP60) capsid (n = 47) and nonstructural (n = 44) and VP60 capsid (n = 47) genes of RHDV strains from Australia and reference sequences. Maximum likelihood phylogenies of the A) VP60 capsid genes and B) nonstructural genes were prepared from an alignment of the newly sequenced RHDV samples (bold) along with published sequences (accession numbers of published sequences indicated in the taxa name). The JN165235/FP/China/2009 and JN165234/XA/China/2010 sequences were restricted to the capsid gene tree because nonstructural gene sequences are not available for these viruses. Variant names for each cluster are indicated. Recombinant (rec) variants are labeled as nonstructural/capsid gene type. Phylogenies were rooted using an early European brown hare syndrome virus isolate (not shown). Bootstrap support values are shown for the major nodes. Scale bars indicate nucleotide substitutions per site. RCV, rabbit calicivirus; RHDV, rabbit hemorrhagic disease virus.

